# A Lecture on Hematemesis; or Vomiting of Blood

**Published:** 1839-03-16

**Authors:** N. Chapman

**Affiliations:** Professor of the Theory and Practice of Physic, in the University of Pennsylvania


### MEDICAL EXAMINER.

DEVOTED TO MEDICINE, SURGERY, AND THE COLLATERAL SCIENCES.

No. 11.] PHILADELPHIA, SATURDAY, MARCH 16, 1839. [Vol. II.

### A LECTURE ON HEMATEMESIS; OR VOMITING OF BLOOD.

*By* N. Chapman, M. D., *Professor of the Theory and Practice of Physic, in the University of Pennsylvania.*

(Concluded from page 150.)

Diversified in its sources, many causes produce vomiting of blood, from which, however, are to be excluded acts of violence, and all others by which vessels are ruptured, such not appertaining to vital hæmorrhage. Even this differs in several particulars which, perhaps, may deserve to be noticed. Females are most liable to it from the commencement to the termination of menstruation, and males at a more advanced period of life. The former have it chiefly as an acute, and the latter as a chronic affection. Connected in the one with a plethoric condition and florid aspect, it is exactly the reverse as to the other,—the appearances of leucophlegmasia, with a shattered constitution, being presented. Exceptions, however, are common to each of these general rules.

Gastric hematemesis, when of an acute and primary character, is excited by circumstances acting directly or indirectly on the stomach,—among the former, are certain acrid, harsh, or stimulating ingesta,—and of the latter, the influence of cold, and other circumstances of a general nature concentrating their force on that organ, and constituting it a centre of fluxion. Being chronic, its production is referrible mainly to those agencies to which gastritis or dyspepsia is assigned. These I shall not now recite, having to do it in detail, on a future occasion, when treating of those subjects.

Of the other varieties, it may be remarked, that the enteric is induced very much in the same way as the gastric,—those of the liver and spleen, by whatever causes congestion or more permanent obstructions of these viscera,—and the vicarious, by a metastasis from the hæmorrhoidal vessels, or the uterus, or some other organ, by which the hæmorrhagic irritation is transferred.

From the history I have given, it may be inferred that it is not always easy to discriminate between the several localities of this hæmorrhage. Careful attention, however, to the more characteristic symptoms of each, will usually conduct us to a correct decision. More readily may it be distinguished from hæmoptysis, which it sometimes resembles. It is seldom preceded, or attended by any pulmonary affection. No cough, dyspnœa, or thoracic pain exists, and the blood is often mixed with the ingesta of the stomach, and is brought up by vomiting. Nearly always it is, also, of a dark colour,—while that in hæmoptysis of the mucous membrane is so only in those rare instances, where, owing to excessive bronchial secretions, an imperfect decarbonization of the blood takes place, from the atmosphere inspired not fully reaching the air-cells.

Greater ambiguity will be experienced in the hæmorrhage incident to pulmonary apoplexy, from the darkness of the blood, and its being ejected in many instances by a sort of convulsive effort, somewhat imitative of vomiting. But here, among other peculiarities, there is extreme thoracic distress, which is very distinctive.

In its ordinary result, vomiting or purging of blood, when copious, is of serious import, as well from the immediate exhaustion induced by it, as from its denoting certain organic lesions, or a general state of system, not very manageable. There are, however, degrees of danger in these several occurrences. Caused merely by a turgescency of vessels, the gastric hæmorrhage is alarming,—and still more so, if connected with any organic lesion of the stomach. Not less applicable are these remarks to that of the bowels. An effusion from the liver or spleen, as being mostly dependent on great derangement, is very apt to prove fatal. Chiefly are recoveries from simple sanguineous congestion of these organs. Effusions of florid, may be held far more favourable, than of dark blood,—the one arising from a feeble, and the other an active condition, and the first being small in quantity.

Examinations, *post-mortem*, show, in the very active, acute, gastric, and enteric hæmorrhage, the mucous membrane of the stomach or bowels, or sometimes both, with diffused floridness, or streaked, or stellated, or its vessels only injected. But such appearances have been seldom noticed, and probably are of very rare occurrence. An infiltration, which is sometimes so thorough that no washing removes the discolouration, may be mistaken, too, for the redness of phlogosis. Much more commonly, it presents a darkish hue, from venous congestion, with patches of ecchymosis, though frequently otherwise, or of nearly its natural complexion, the vessels being emptied by the escape of the blood. Lesions of a chronic character are also to be met with, sometimes a varicose state of the veins, or engorgement, or inflammation, in every stage of its progress,—softening or induration of texture, thickening or the reverse, common ulceration, or scirrhosity and open cancer. But some of these phenomena can hardly be considered as belonging to vital hæmorrhage. The liver and spleen, when they are the seat of the hæmorrhage, are found in every variety of condition, either mere engorgement, or the several grades of disorganization of structure, to the extremest extent.

Of the pathology of this hæmorrhage, it is to be observed, in the first place, that its occurrences, when very profuse, are generally of an inactive nature. The blood in the gastric and enteritic cases comes from the exhalents of the mucous membrane,—and in the hepatic and splenitic, it is supposed to proceed from those of the interstitial tissue, passing out in the former instance through the ductus cholidicus, and in the latter from the extremities of the vasa brevia into the stomach,—neither of which is probable. Granting, however, that such may be the occasional modes of its escape, it appears to me very clearly that the effusion generally, and, perhaps, always, excepting where there is sluggish congestion of these viscera, issues out of the mucous membrane of the alimentary canal, caused by an irritation derived from the diseased organs, exactly as they influence the production of serous and cellular dropsies,—of which more hereafter.

Commencing the treatment of hematemesis with that of the stomach, I shall subsequently indicate the modifications of it required by the other varieties of the disease. Called to a case, however induced, with any activity of pulse, or warmth of surface, venesection becomes proper, to be followed, or substituted, according to the severity of the attack, by cups or leeches, and a sinapism or blister to the epigastrium. Consulted in time, these remedies, with low diet, and rest, will very often prevent the hæmorrhage. To check the flow of blood, the coldest water, or the swallowing of ice itself, and cold applications over the stomach, are useful. To the same end various astringents are employed, as a solution of common salt, or alum, or sugar of lead, or white vitriol, or the muriated tincture of iron, or the sulphuric or gallic acid, or the creosote, or the spirit of turpentine. Excepting the last, which is undoubtedly serviceable, all the rest are, in my opinion, very equivocal medicines. Even the turpentine should not be prescribed when any activity of inflammation is suspected.

Emetics, in the absence of phlogosis, are entitled to the largest share of confidence, and have been prescribed by me with signal advantage. To the use of them I was led by the views I entertain of the pathology of hæmorrhage, and particularly of their influence over the capillaries. More than any other process does vomiting, from ipecacuanha especially, change that condition of the exhalents which favours sanguineous effusions,—though, in this case, not a little, also, is to be ascribed to its removal of large clots of blood by which the stomach is oppressed. Nor should we be unduly intimidated, or discouraged from a resort to an emetic, by the state of the pulse, or general feebleness. This is a condition commonly to be expected,—and so far as I have seen, the recuperative energies are revived by the operation of the remedy, and especially when a large amount of blood is voided.

Two cases, out of a number in my possession, I shall select, to exemplify the safety and utility of this practice.

In 1818, I had under my care a girl of eighteen years of age, of a leucophlegmatic temperament, exceedingly distressed by the train of dyspeptic symptoms already enumerated, who, while under the common treatment for such affections, was in the night attacked with a vomiting of blood. On my visiting her, I learned that in less than an hour she had thrown up about three pints, and the hæmorrhage continued after I saw her, till nearly one pint more was discharged. The usual astringent remedies were unavailingly tried,—and as the exhaustion had become so extreme as to menace speedy dissolution, I resolved on the use of an emetic, encouraged by my experience of its success in some former cases of less violence, and ipecacuanha was accordingly exhibited very freely. Ejecting a large quantity of dark grumous blood, she soon after became composed,—her pulse rose, the skin resumed its warmth, and before morning, I left her doing well in every respect. No return of the hæmorrhage took place on this occasion,—and in a few weeks she went into the country, where I understand she completely recovered.

By a lady, who consulted me in 1827, I was informed, that from the cessation of her menses some six months previously, she had been much afflicted by headache, burning sensations in the stomach, præcordial uneasiness, tension and tumidity of the epigastrium, nausea, and periodical vomitings of small portions of blood.

Her appearance, at this time, was valetudinary,—and on the investigation of the case, I was confirmed in the suspicion I at once adopted, that if not arrested, it must inevitably lead to a serious attack of hematemesis. But she was prepared to take a short journey,—and, perhaps, confiding more in exercise and fresh air than in medical prescriptions, it was agreed that they should be postponed till her return to the city. Ten days afterwards my prediction was verified, for in getting out of a carriage she was seized with a vomiting of blood, repeated at short intervals, till the whole amounted to several pints. Her pulse being active, the skin tolerably warm, and some sensibility of the epigastrium existing, the treatment was commenced by leeches, followed by cold applications over the stomach, and small portions of cold acidulated drinks. No advantage resulted from these measures, and debility becoming alarming, she at length consented to take an emetic, which evacuated some considerable masses of blood, and for several hours she was greatly relieved. The vomiting, however, again recurring, I had to repeat the emetic, which proved very effectual. Convalescence henceforward took place, and by a properly regulated regimen chiefly, her health was pretty well re-established.

Not much is to be found of this practice in hematemesis. Excepting, indeed, some cases very analogous to those I have related, which were successfully treated, in precisely the same way, by Dr. Sheridan, contained in a late volume of the Dublin College of Physicians, I have been unable to discover any notices of it.

Concerning the other varieties of this hæmorrhage, I have to state, that the treatment in the early stage of each is essentially the same, adapted to the condition of the system, and to the part affected. Thus, in sudden and active congestions, or phlogosis of the bowels, liver, or spleen, we resort to venesection, or leech, or cup and blister the centre, or the right or left side of the abdomen, according to the indication. Cold applications, even of ice, are here, also, sometimes very beneficial.

Cases, however, of any activity of condition, and particularly those of the viscera of the hypochondriac regions, are seldom met with, and the detraction of blood, generally or locally, is hence inadmissible. Cold appliances, too, seem to do harm, by increasing the torpor of the venous circulation, and thereby aggravating the already existing congestion. Blisters and sinapisms I have found nugatory. The effect of an emetic, under such circumstances, I cannot say, having no experience with it. From its known properties, and its decided utility in analogous instances, might it not be ventured in an emergency, and more especially, as our resources are so limited and precarious?

On the whole, I have derived the most constant and unequivocal advantage in all these cases, including the active states of the hæmorrhage, on a proper reduction by depletion, from the liberal employment of the spirits of turpentine. More than thirty years have I prescribed it, and am prepared to affirm that it is deserving of greater confidence than any other known to me. The same favourable opinion is entertained of it by many of my medical friends,—and I have learned that Dr. Broeke, of Dublin, a very distinguished practitioner, has lately reported several cases of melæna treated successfully by it. My mode of giving it, is the dose of from twenty drops to a drachm, repeated more or less frequently, according to the exigency.

Being loaded and distended, it is urgently required to evacuate the bowels. This condition is nearly always productive of extreme depression of the vital powers, evinced by feeble circulation, cold skin, heavy, anxious breathing, and nausea, or vomiting,—all which may probably be relieved by the removal of large quantities of grumous blood. Castor oil, with a portion of the spirits of turpentine added to it, I deem to be best adapted to the purpose. Even should no such accumulation exist, purging is useful, and cannot be safely pretermitted, where constipation, or a tendency to it prevails. It is, indeed, said by the celebrated Hamilton, that a species of hematemesis, occurring in females in early life, is promptly cured by it only. That this particular hæmorrhage, which is proved to have no connexion with any structural injury of the stomach, is a discharge vicarious of the menses, had long been maintained,—and such is still my opinion, since, among other reasons sustaining it, I have remarked in the cases coming under my notice, either a retention or suppression of the menses.

Transiently, it may be mentioned, that effusions of blood from some part are very apt to occur on a sudden suppression of the catamenia,—of which a case is related by Hufeland, of a woman, who, having the discharge checked by taking cold, fell sick the same evening, and the next morning died. On inspection, three pints of blood were found in the peritoneal cavity, without, however, any inflammation or other lesions, in any portion of the body.*

An instance very similar happened in my own practice,—that of a girl, who, having imprudently gone into a cold bath while menstruating, had the discharge stopped, and soon after was seized with raving delirium, of which she died the next day,—and on opening the cranium, large quantities of extravasated blood were observed in several portions of the brain.

Hamilton, however, contends, that the sort of hematemesis he alludes to, proceeds from, or at least is mainly dependent on, a constipated state of the bowels, the fæces brought off being always copious, and of an unnatural colour, consistence, and smell. The success of this, compared with the former mode of treatment, I will not take on myself positively to pronounce on,—though I am inclined to believe that purgatives have been too sparingly used in such cases. Certain it is, that in chlorosis, so intimately associated with the hæmorrhage before us, they are among our most efficacious remedies. Governed, however, by the view which I have expressed of the pathology of the case, it being essentially vicarious, it has been managed by me accordingly, having, as a principal object, the establishment or restoration of the uterine function.

As to diet in these hæmorrhages, some discrimination is demanded. During the flow of blood in the active form of them, cold, mucilaginous drinks, acidulated, or ice water, or ice itself, in small portions, should only be allowed. But in an opposite state, and especially under circumstances of exhaustion, which often happen, more cordial and stimulating beverages become proper, such as wine, or even ardent spirits diluted. On the admission of food, care must be observed that it be of the mildest kind, and very little taken at a time. This precept, of general excellence, is especially so, as regards the affections of the stomach and upper bowels.

To check, merely, however, this or any other hæmorrhage, is partially to perform our duty. It remains, still, to guard against recurrences, which demands the ascertainment of the condition giving rise to it, and the institution of an appropriate treatment for its removal. Considering how often are the abdominal hæmorrhages inseparable from the most serious lesions of the viscera, such a course seems to be imperatively dictated.

---

### CASE OF POLYPUS OF THE VULVA.

By David M. Wright, M. D.

*January 17th.*—I was called a few miles in the country to visit a negro girl, about 16 years of age, who was represented to be in a very perilous

* Philadelphia Journal, vol. vii., p. 182.

